# Addressing the gap: a blueprint for studying bimanual hand preference in infants

**DOI:** 10.3389/fpsyg.2015.00560

**Published:** 2015-05-05

**Authors:** Sandy L. Gonzalez, Eliza L. Nelson

**Affiliations:** Department of Psychology, Florida International UniversityMiami, FL, USA

**Keywords:** handedness, hand preference, bimanual manipulation, infants, toddlers

## What is role-differentiated bimanual manipulation?

Between 4 and 7 months of age, infants begin to manipulate objects using role-differentiated bimanual manipulation (RDBM) where one hand stabilizes an object while the other hand manipulates the object (Rochat, 1989; Kimmerle et al., 1995, 2010). Because RDBM constrains the roles of the hands, it elicits a *measurable asymmetry* where the manipulating hand is considered to be the preferred hand for RDBM actions (i.e., RDBM hand preference). Initially, infants display partially differentiated roles for each hand, which is driven in part by the affordances of the object (Ramsay et al., 1979; Fagard and Jacquet, 1989; Fagard and Pezé, 1997; Fagard, 1998; Fagard and Marks, 2000). Children exhibit increasing role differentiation with age (Vauclair and Imbault, 2009; Birtles et al., 2011; Cochet et al., 2011; Cochet, 2012). Only 50% of infants' bimanual actions were characterized as fully differentiated at 12–13 months (Ramsay and Weber, 1986). At 18 months, children used a fully differentiated strategy on 71% of target RDBM actions; this figure increased to 94% by 24 months (Nelson et al., 2013). Early emerging RDBM skills (11–13 months) involve object removal and insertion, while later RDBM skills (18–24 months) include additional actions such as unscrewing and unzipping (e.g., Kimmerle et al., 2010; Nelson et al., 2013).

## The development of RDBM hand preference

Longitudinal studies have found that RDBM hand preference emerges around 11–13 months with a trend favoring the right hand (Michel et al., 1985; Kimmerle et al., 1995, 2010). A cross-sectional study on infants aged 10–17 months similarly reported a right trend in roughly half of the sample (Vauclair and Imbault, 2009). Additional work during the period of role differentiation development has been limited. In a small sample of eight participants, Cochet (2012) reported that one child was consistent in right RDBM hand preference across six visits from 15 to 25 months, whereas the other children were variable and primarily shifted between exhibiting a right hand preference and no hand preference. Similarly, Cochet et al. (2011) reported a right trend for RDBM hand preference from 14 to 20 months. However, individual trajectories were not analyzed due to the nature of their design.

In a study that examined individual trajectories in a larger sample of 38 children, Nelson et al. (2013) found 76% of children exhibited a *consistent right* RDBM hand preference for 7 monthly visits from 18 to 24 months, while 21% exhibited a *consistent left* RDBM hand preference. Only one child could not be classified as having a statistically significant hand preference for RDBM. Further preliminary examination using latent class growth analysis in this expanded data set found that trajectories for RDBM hand preference are flat (i.e., linear slopes are 0), suggesting that hand preference for RDBM may be established by 18 months (Gonzalez et al., 2015). Taken together, RDBM hand preference is hypothesized to develop between 11 and 18 months.

## The gap in RBDM knowledge

To date, no longitudinal study has incorporated the complete hypothesized age range of RDBM hand preference development from 11 to 18 months, creating a gap in our knowledge. Prior work has provided valuable information about the general timing and patterning of RDBM hand preference, but does not provide sufficient detail regarding individual differences. The variability previously reported by multiple investigators suggests equifinality in handedness—although 90% of adults are right-handed (e.g., Annett, 2002), there may be multiple trajectories toward this endpoint. Without adequate characterization of the trajectories for RDBM hand preference, investigators cannot accurately address the developmental relationship between hand preference and other neurocognitive asymmetries, such as language.

## Addressing the gap: a modest blueprint

The use of trajectory-based longitudinal designs is the key to addressing the gap in our knowledge of RDBM hand preference. Trajectory-based longitudinal methods allow researchers to find commonalities between subjects when measured at regular intervals using latent class growth analysis or a related appropriate statistical approach. Based on common patterns, subgroups of participants are identified, with each subgroup demonstrating a distinct trajectory. Individuals in one trajectory group are more similar to each other than they are to individuals of another trajectory group. Importantly, group-based trajectories accommodate variability in hand use seen at individual time points: although there may be some fluctuation across time points, a group-based trajectory approach can identify overall robust patterns of hand preference (Michel et al., 2014; Campbell et al., 2015).

Recent use of these methods to investigate hand preference for acquiring objects identified three distinct hand preference trajectories in a sample of 328 infants followed longitudinally from 6 to 14 months: infants with a right hand preference, a left hand preference, or no hand preference (Michel et al., 2014). By adopting a design that spans the period when hand preference for acquiring objects emerges and focusing on identifying distinct group patterns over time, Michel and colleagues have countered the notion that hand preference is unstable in infancy (e.g., McManus et al., 1988; Corbetta and Thelen, 1999). The impression of instability in hand preference may in fact stem from the use of cross-sectional designs as well as small sample sizes that prevent the identification of group-based trajectory patterns.

Using the same trajectory-based longitudinal methods described for infant object acquisition, researchers can begin to parse trajectories for RDBM hand preference. In the following sections, we provide suggestions for how to effectively design future research studies on the development of RDBM hand preference within the framework of trajectory-based longitudinal methods. Our suggestions are guided by recent work on hand preference for acquiring objects in infants, as well as recommendations from comparative non-human primate studies. By providing these guidelines, we hope to encourage research that might fill the gap in our knowledge of how RDBM hand preference develops, how RDBM relates to other hand use and cognitive skills, and to begin a conversation on standardizing methodology to promote further crosstalk between disciplines.

### Recommendation #1: measuring the development of (RDBM) hand preference requires longitudinal designs

In order to properly characterize hand preference during development, researchers must employ longitudinal designs. Such designs permit the emergence of a skill, and subsequently a hand preference for that skill, to be adequately measured and participants appropriately classified into the correct trajectory. For RDBM, we recommend that future research address the span of 11–18 months, which corresponds to prior longitudinal observations of RDBM hand preference development using subsets of this range. Crucially, when implementing trajectory-based longitudinal methods, assessment of RDBM hand preference must be done on at least a *monthly* basis. By systematically adjusting the number of time points used for latent class analysis (up to 9 monthly assessments), Michel et al. (2014) found that fewer months resulted in fewer trajectories. Measurements from only odd months (7, 9, 11, 13) identified only two groups: infants with a right preference or no hand preference. Conversely, measurements from even months (6, 8, 10, 12, 14) identified two different groups: infants with a right preference or infants with a left preference. Thus, measuring hand preference at only some months of the age range in question can result in different hand preference classifications.

Use of only 1 month (i.e., single time point) to classify hand preference is equally problematic. For example, use of only the 12-month-old score resulted in misclassification of about 40% of infants' hand preferences when compared to their trajectory from 6 to 14 months (Michel et al., 2014). Selecting other single months from the trajectory resulted in similar or increased percentages of misclassification, putting into question the statistical reliability of single month assessment used in cross-sectional studies (Michel et al., 2014). Hence, it is imperative that future work utilizes longitudinal methods across the hypothesized period of RDBM hand preference (11–18 months) to accurately identify developmental trajectories.

### Recommendation #2: measuring the development of (RDBM) hand preference requires sufficient trial numbers for statistical analysis

When measuring hand preference, researchers should be cognizant of the amount of trials administered to participants; a sufficient number of trials are needed for a robust *statistical* identification of hand preference. In past research, the total number of trials given for RDBM assessments has varied widely, ranging from 2 to 29 (e.g., Fagard and Lockman, 2005; Nelson et al., 2013). Based on recent trajectory-based findings comparing trial number for infant object acquisition hand preference (Campbell et al., 2015), and suggestions from non-human primate research (Hopkins, 2013a,b), we advocate for the use of *at least* 25 trials when measuring RDBM hand preference in any target population.

Campbell et al. (2015) compared two major infant hand preference assessment batteries in a sample of 150 infants across 7 monthly assessments from 8 to 14 months: one with nine trials and another with 32 trials. Statistically, eight or more lateralized responses out of nine are needed for significance using the binomial test on the smaller test battery. However, arbitrary proportion cutoffs are often used instead to classify participants. A common proportion formula is the Handedness Index: *HI*=[(R−L)/(R+L)] (where *R* indicates number of right hand responses, *L* indicates number of left hand responses). Bimanual responses can also be included in the denominator if the action permits them (e.g., *HI*=[(R−L)/(R+L+B)]). HI varies continuously from −1.00 to 1.00 with negative values interpreted as leftward and positive values interpreted as rightward. Trajectory-based analyses revealed that the 9-trial battery only identified right preference infants and infants with no hand preference. Conversely, the 32-trial battery identified the three trajectory groups previously found by Michel et al. (2014) in a larger sample of 328 infants: right preference, left preference, and no preference (Campbell et al., 2015).

Notably, administering sufficient trials allows researchers to conduct more robust statistical tests to determine hand preference. A minimum of 25 trials is required if researchers use a z-score transformation of the HI. A z-score transformation determines hand preference based on statistical probability by using z-score critical values of ±1.96. If researchers prefer to use HI scores, administration of 30 trials allows for use of −0.20 and + 0.20 as z-score equivalent cut-off points (Hopkins, 2013b). Using other HI cut-off points such as ±0.50 may underestimate hand preference, even with more than 30 trials, labeling most children as having no preference (Campbell et al., 2015). The use of z-score transformations or HI ± 0.20 is advantageous, as both preclude the need to determine arbitrary HI cut-off points, which are not uniform across handedness literature in either human or non-human studies (for discussion, see Hopkins, 2013a,b).

## Conclusion

Promising findings from trajectory-based longitudinal studies on hand preference for object acquisition indicate that distinct trajectories for hand preference are identifiable in infancy (Michel et al., 2014; Campbell et al., 2015). We urge researchers interested in the development of RDBM hand preference to adopt similar trajectory-based methods in an effort to fill the gap in our knowledge for how and when RDBM hand preference develops. To accurately capture developmental trajectories, we recommend that future research measure RDBM hand preference at least monthly from 11 to 18 months of age using longitudinal designs, and utilizing at least 25 trials in the test battery (Hopkins, 2013b; Michel et al., 2014; Campbell et al., 2015). Not addressed here is whether preference trajectories are sensitive to varying RDBM task demands (e.g., object removal vs. unzipping) or the types of objects used in assessments. Both of these variables merit further investigation.

Characterizing hand preference trajectories for RDBM is an important step toward understanding how experience-dependent changes in motor skills relate to gains in other domains. Research on hand use preference for an earlier motor skill (i.e., object acquisition) has demonstrated that left and right preference trajectories do not mirror each other in development, and infants with no hand preference also display a separate and distinct trajectory (Michel et al., 2014). Moreover, an early right preference for infant object acquisition was associated with advanced language skills at 2 years of age (Nelson et al., 2014). Differences in hand preference trajectories may relate to differential outcomes in other cognitive domains. Thus, it is imperative that future studies utilize a trajectory-based approach that statistically characterizes infants and toddlers into distinct hand use patterns. Doing so will propel the field toward bridging another gap: understanding the role of handedness in neurobehavioral development.

### Conflict of interest statement

The authors declare that the research was conducted in the absence of any commercial or financial relationships that could be construed as a potential conflict of interest.
